# Managing anxiety-related disorders from pregnancy to parenthood

**DOI:** 10.1007/s00404-026-08377-4

**Published:** 2026-03-10

**Authors:** Willemijn Scholten, Ilja Saris, Eline Eigenhuis, Lisa de Koning, Anna Muntingh, Bibi Schut, Adrie Seldenrijk, Patricia van Oppen, Neeltje Batelaan

**Affiliations:** 1 Outpatient Clinic for Anxiety and Obsessive-Compulsive Disorders, GGZ inGeest, Amsterdam, The Netherlands; 2 Department of Psychiatry, Amsterdam UMC, Amsterdam, The Netherlands; 3 Amsterdam Public Health Research Institute, Mental Health Program, Amsterdam, The Netherlands

**Keywords:** Anxiety disorders, PTSD, OCD, Pregnancy, Parenthood, Antidepressants, Psychotherapy, Exposure therapy

## Abstract

**Purpose:**

Anxiety-related disorders (ARD), including posttraumatic stress disorder (PTSD) and obsessive–compulsive disorder (OCD), are highly prevalent mental health conditions. The peak prevalence of ARD in women coincides with the critical period of family planning, pregnancy, and child-rearing, affecting 1 in 5 women. This poses several challenges, including fears of passing anxiety on to children, worsening of symptoms during pregnancy or postpartum, and concerns about how therapy affects pregnancy outcomes. Clinicians frequently lack the expertise to adequately address these concerns. This may result in clinicians being cautious about starting treatment. This narrative review provides insights from the literature along with practical recommendations to facilitate decision-making with these challenges.

**Methods:**

This narrative review provides a review of existing literature on ARD and pregnancy, synthesizing key findings from relevant theoretical and empirical studies.

**Results:**

Results show that ARD tend to cluster within families, driven by both genetic and environmental factors. During pregnancy and postpartum, ARD are particularly prevalent, and maternal anxiety is associated with an increased risk of preterm birth and low birth weight. Psychotherapy, including exposure therapy, is effective and is overall beneficial for pregnant women, although in specific cases, it can also worsen the anxiety, with no known adverse effects on pregnancy outcomes. SSRI use requires consideration of risks and benefits. Preventive strategies to reduce anxiety vulnerability in offspring are scarce.

**Conclusion:**

In conclusion, addressing ARD in (prospective) parents is essential, given the potential negative impact on both parents and children. Clinical awareness is needed to optimize care for this population.

## What does this study add to the clinical work

**Table:** 

This research highlights the intersection of anxiety-related disorders (ARD) with critical reproductive periods, underscoring the increased prevalence and familial transmission risks of ARD during family planning, pregnancy, and child-rearing.	
It is particularly relevant for multidisciplinary teams involved in the treatment of pregnant patients with anxiety, as it enhances understanding of the impact on pregnancy outcomes and child development.	
The findings emphasize the efficacy of psychotherapy and the careful management of SSRIs, offering practical recommendations for treatment approaches that consider both parental and child health. This knowledge supports clinicians in making informed decisions, potentially improving long-term outcomes for families affected by ARD.	

## Introduction

Anxiety-related disorders (ARD) are among the most common mental disorders in the general population, and include DSM-5 anxiety disorders, posttraumatic stress disorder (PTSD), and obsessive–compulsive disorder (OCD) [1]. While anxiety disorders refer specifically to diagnoses, such as generalized anxiety disorder (GAD), panic disorder (PD), and social anxiety disorder (SAD), ARD, an umbrella term, also includes posttraumatic stress disorder (PTSD) and obsessive–compulsive disorder (OCD), which are classified separately in the DSM-5 but share common etiological, phenomenological, and treatment features. In the perinatal context, the term *perinatal anxiety* is sometimes used even more broadly, capturing both formally diagnosed anxiety disorders and anxiety symptoms that may not meet full diagnostic criteria but are nevertheless clinically relevant.

The peak prevalence occurs in the child-bearing and child-rearing age [2]. Current and prospective parents with an ARD struggle with questions and concerns about heredity and progression of symptoms during pregnancy and in the postpartum period. They also worry about the adverse effects of medication usage and psychotherapy on pregnancy outcomes, and about the effect of their own anxiety on their offspring during child-rearing [3]. Clinicians involved in guiding patients with ARD in the perinatal period, such as psychiatrists, psychologists, and gynecologists, frequently lack sufficient knowledge to adequately inform prospective parents, or they may be hesitant in initiating psychotherapy or pharmacotherapy [3]. We provide insights from the literature and give practical recommendations for clinicians regarding the prevalence, risks, treatment options, and preventive strategies for current and future parents with ARD. This review builds on existing rigorous systematic reviews and guidelines [4–6] of anxiety disorders in the perinatal period by integrating findings and expanding the scope to a broad, clinically oriented overview, including the epidemiological findings, obstetric outcomes, intergenerational transmission, and the safety and effectiveness of psychotherapy and SSRI treatment. Our aim is to enhance clinicians' knowledge, enabling them to inform their patients and guide clinical decision-making. Consequently, patients should be enabled to make well-informed choices about parenthood, receive proper treatment, and obtain tools to reduce the risk of ARD in their children. This narrative review was conducted to provide a broad, clinically oriented overview of anxiety-related disorders (ARD) in the context of family planning, pregnancy, the postpartum period, and early parenthood. Our aim was not to produce a comprehensive systematic review, but rather to synthesize key findings and offer practical guidance for clinicians involved in perinatal mental healthcare.

## Methods

An initial literature search was performed primarily in PubMed using core search terms aligned with the main topics of the review (e.g., perinatal anxiety, pregnancy, postpartum, anxiety disorders, SSRI, psychotherapy, cognitive behavioral therapy, exposure therapy, EMDR, PTSD, and OCD). Searches were organized per section to ensure coverage of epidemiology, obstetric outcomes, intergenerational transmission, and treatment approaches. We subsequently used a snowballing strategy by screening reference lists of relevant articles and guidelines to identify additional sources.

Inclusion criteria were: (1) human studies, (2) publications in English or Dutch, (3) a focus on the perinatal period (preconception to early parenthood), and (4) studies published within the past 25 years. We prioritized meta-analyses, systematic reviews, large cohort studies, and clinical guidelines. Exclusion criteria included case reports and studies with very small sample sizes.

To ensure the use of high-quality evidence, we relied on existing risk-of-bias assessments reported in major meta-analyses and reviews and applied a simplified appraisal of methodological rigor for key cohort studies (e.g., sample size, diagnostic criteria, and outcome measures).

### Prevalence, onset, and course of ARD during pregnancy and postpartum

Understanding the prevalence and impact of ARD in women and men in transition to parenthood is essential for reducing its potential negative outcomes. ARD are highly prevalent in women during pregnancy and in the postpartum period. About 20% of women meet criteria for at least one ARD during pregnancy or the postpartum period [7], suggesting a significantly increased risk, as compared with the global year-prevalence rate of 8.7% for ARD found in women (Table 1) [1]. Correspondingly, a direct comparison in a Turkish case–control study (*n* = 1482, *N* = 1154 pregnant women, with *N* = 328 pregnant premenopausal controls) has shown that pregnant women have an increased risk of having at least two ARD [8]. In addition, another cohort study has shown that mothers with prepartum ARD had an increased risk of postpartum depression [9].

**Table 1:** Prevalence of anxiety-related disorders in women during pregnancy and postpartum

	Prevalence rates in % (95% highest density interval)		
Anxiety-related disorder	Pregnancy	Postpartum	Overall peripartum
Panic disorder	1.6 (0.9–2.6)	2.3 (1.1–3.9)	1.9 (1.1–2.8)
Agoraphobia	3.2 (1.3–5.5)	1.0 (0.1–2.7)	2.4 (1.0–4.0)
Obsessive–compulsive disorder	2.3 (1.1–3.9)	1.7 (0.2–4.3)	2.2 (1.2–3.6)
Generalized anxiety disorder	2.0 (1.1–3.4)	2.6 (1.2–4.3)	2.4 (1.3–3.8)
Social phobia	2.4 (1.4–3.5)	2.3 (1.2–3.7)	2.4 (1.6–3.5)
Specific phobia	4.3 (1.9–7.0)	4.6 (2.0–7.7)	4.8 (2.4–7.7)
PTSD	1.3 (0.5–2.4)	0.3 (0.0–1.4)	1.1 (0.5–2.0)
Anxiety not otherwise specified	2.6 (0.8–5.0)	0.8 (0.0–4.6)	2.3 (0.5–4.5)

Prevalence estimates from a study by Fawcett et al. 2019, figs. 2 and 3 [7]

Although information about the course of ARD during pregnancy should be viewed with caution, primarily due to the limited number of studies and substantial variability in pooled estimates, available studies do point in the same direction: toward increased vulnerability for ARD during pregnancy. In a recent, thorough review, the course of ARD was studied. Feldman and colleagues (2025) estimated a global prevalence of 12.3% for postpartum anxiety disorders, taking into account study sample sizes and including worldwide studies [10].

Interestingly, evidence suggests that transition into parenthood may also increase the risk for anxiety in men. According to Leiferman and colleagues, 10.7% of the fathers experienced perinatal anxiety (symptoms or clinical diagnoses) [11]. This rate appears to be considerably higher than the global WHO prevalence rates for anxiety among men, which range from 2.2 to 3.8% [12]. Unfortunately, research on ARD in (prospective) fathers is scarce, limiting our understanding.

### Pregnancy outcomes

Globally, preterm birth occurs (PTB) in approximately 11% of deliveries, while low birth weight (LBW) is observed in 15% of births [13, 14]. It is important to determine whether maternal anxiety disorders during pregnancy increase these risks, as such knowledge could help clinicians decide whether, and to what extent, pregnant women should undergo anxiety screening and treatment during pregnancy. A meta-analysis (12 studies totaling 17,304 pregnant women reported PTB data; 6 studies totaling 4948 pregnant women reported LBW data) found modest but significant associations between maternal anxiety symptoms during pregnancy and PTB. An association between prenatal anxiety and LBW was found for women from Asian countries, but not in those from European countries (pooled RR = 1.50, 95% CI [1.33, 1.70]), and LBW (pooled RR = 1.76, 95% CI [1.32–2.33]) [15]. A lower socioeconomic status and prenatal care index (a measure of the adequacy of prenatal care) were linked to a higher risk of PTB and LBW. It must be mentioned that the included studies varied largely in settings (for instance, countries, limited access to prenatal care, and malnutrition) and types of anxiety (anxiety symptoms, pregnancy-related anxiety, and anxiety disorders), warranting careful interpretation. A more robust finding is the association between anxiety disorders in pregnancy and hypertensive disorders during pregnancy. Increased presence of hypertension was associated with both pre-pregnancy anxiety and anxiety disorders during pregnancy [16].

### Behavior or neurodevelopmental changes in children

The influence of perinatal ARD in women on behavioral or neurodevelopmental changes in children in early and middle childhood was studied in a systematic review including 47 studies by Quagliato et al. (2022) [17]. Prenatal maternal anxiety and postnatal anxiety appear to be associated with variables, such as negative temperament, decreased alertness, impaired psychomotor and cognitive development in children, and anxiety symptoms from childhood to adolescence. Although the underlying mechanisms and pathways are not yet fully understood, evidence suggests that some of these alterations originate in the uterus [18]. An increase in psychopathology and neurodevelopmental changes in offspring underscores the significance of recognizing family mental health status as a factor in assessing vulnerability to mental health disorders.

### Parental transmission of ARD to children

In addition to heightened risks for adverse pregnancy outcomes, patients, their spouses, and clinicians often wonder about the genetic odds of transmitting ARD. Also, patients question whether their own anxious behavior or parental styles cause ARD in their children. Studies indicate that children of parents with ARD have a two-to-threefold increased risk of developing ARD themselves, alongside a slightly increased likelihood of developing depression [19]. Up to 60% of these children may eventually develop ARD [20]. Two key factors contributing to this increased risk, as highlighted in the literature, are genetics and environmental factors [21–26].

Based on twin studies, genetic factors account for 30–50% of the vulnerability to develop ARD [24]. Although significant, the genetic basis is complex and not fully understood [21, 22]. Most likely, transmission occurs through a complex interplay between genetics and environmental factors [23, 24]. It seems that, in early life, genetic factors explain the majority of variation of ARD expression, while later in life, when children are older, the shared environment is more important in the development of ARD [20, 21].

In terms of parenting styles, some evidence supports non-genetic pathways linking parental behavior and parental styles to anxiety symptoms in offspring during childhood, but evidence is inconclusive [27–30]. Longitudinal data remain insufficient, according to a systematic review on this topic [26].

## Treatment options for parents and their children

### Psychotherapy during pregnancy

Patients with ARD largely prefer psychotherapy over pharmacotherapy. This was shown in a multidisciplinary anxiety disorders clinic in the United States (*n* = 103), where patients with any anxiety or obsessive–compulsive disorder completed the Treatment Perceptions Questionnaire, rating each treatment as first choice, second choice, or not at all. For pregnant women with ARD specifically, psychotherapy as monotherapy is preferred by 74%, as compared to 47% in non-pregnant women [31].

However, there is an ongoing debate about whether psychotherapy, specifically exposure therapy as a first-line treatment in ARD, is safe for the mother and the fetus [32]. Concerns arise from the increased arousal associated with exposure therapy and potential consequences for the fetus. In exposure therapy, patients confront their fears; for instance, individuals with a fear of using public transport may be (gradually) exposed to riding a bus, which often leads to physical arousal. Therapists have expressed reservations about employing exposure therapy for patients with ARD [33]. Research on therapist beliefs regarding the use of exposure therapy specifically for pregnant patients is lacking. However, a study on reasons why clinicians exclude people from exposure therapy showed that a stronger belief in negative perceptions of exposure therapy was significantly linked to a higher likelihood of excluding clients from this treatment [34]. It is likely that therapists would exercise even more caution when applying exposure to this particular subgroup of pregnant women. Pregnant women are excluded from most large CBT studies on ARD, likely at least in part due to concerns about potential risks associated with exposure therapy during pregnancy. Fortunately, a few studies on the effectiveness of CBT, including exposure therapy, have been conducted in pregnant women, and these show significant reductions in, or prevention of symptoms of anxiety [35–38]. By synthesizing evidence from 79 randomized and quasi-randomized trials on anxiety and depression, a recent meta-analysis found that CBT provided both short- and long-term reductions in perinatal anxiety (different disorders were not specified), with moderate effect sizes [38]. In another study, an intensive 2-week exposure program showed to be feasible and effective in pregnant women with ARD [39].

#### Safety of psychotherapy for the unborn child

Research shows no evidence that psychotherapy (including exposure therapy) harms the unborn child or affects child-related outcomes (e.g., Apgar scores, birth weight, and gestational age [39–42]). A meta-analysis of 16 RCTs (n = 2778) found no significant effects of prenatal psychological or other non-pharmacological interventions on birth weight, Apgar scores, or gestational age; given the generally high risk of bias in the included trials, these results should be interpreted with caution, and evidence remains insufficient to support benefits of antenatal treatments on offspring outcomes [41]. In an RCT treating mothers experiencing fear of childbirth (FoC), Eye Movement Desensitization and Reprocessing (EMDR) was compared to treatment-as-usual. Although EMDR was shown to be effective and safe, interestingly, FoC appeared self-limiting [43].

Overall, these studies show that psychotherapy for pregnant women seems effective, and there are no indications of adverse events for the fetus. It should be noted, however, that it is difficult to study adverse effects with a very low incidence, so potential negative effects cannot be ruled out completely. There are also insufficient data to substantiate any beneficial effects on child-related outcome measures.

### Pharmacotherapy around pregnancy

In clinical practice, one of the most urgent concerns for patients with ARD is the safety of medication use during pregnancy or breastfeeding. Key questions include the impact of selective serotonin reuptake inhibitors (SSRIs) on fertility, the safety of continuing or starting SSRIs during pregnancy, and recommendations for breastfeeding [3]. Thankfully, the body of evidence on this topic is growing. In line with exposure therapy, SSRIs are a first-line treatment for ARD. Approximately 6% of pregnant women in the USA used SSRIs between 1995 and 2006 [44, 45].

#### Conception and fertility

SSRI use appears to have no significant impact on female fertility [46]. The impact of SSRI use on male fertility remains inconclusive. SSRI use in the 3 months before conception may reduce sperm concentration, motility, and shape [46–48], though some studies have reported no effect [49, 50]. If fertility problems and reduced sperm quality are evident, tapering off the SSRI might be considered. There is no proven risk of birth defects in children of men using SSRI in the 3 months before conception [51].

#### Risks of maternal SSRI use during pregnancy for the fetus

As mentioned above, ARD itself are tentatively linked to obstetric and neonatal complications, such as preterm birth and low birth weight. These associations were irrespective of SSRI use, although women using SSRIs seemed to carry a slightly higher risk of these complications [45, 52–54].

The link between SSRI use during pregnancy and congenital cardiac malformations is extensively studied. Previous findings, associating paroxetine in the first trimester with cardiac malformations, were not confirmed by more recent and better controlled studies [45, 54]. It is known that newborns exposed to SSRIs during late pregnancy have a higher risk of persistent pulmonary hypertension (PPH). PPH is a serious condition with high morbidity and mortality [45, 55], but has a low incidence (2.9 per 1000 live births [55]). Sertraline is most likely the SSRI with the lowest risk for PPH of the newborn, as compared to other frequently used SSRIs (escitalopram, paroxetine, citalopram, and fluoxetine) [56].

#### Risks of SSRI use during pregnancy for the mother

Risks are also present for the mother using SSRIs. The risk of both postpartum hemorrhage and pre-eclampsia is dose-dependent, although the relative risk remains small [45, 57].

In the decision regarding SSRI continuation during pregnancy, the risk of relapse after tapering off medication must be weighed against the risks of continued SSRI use for both mother and child. Compared to continuing antidepressants, discontinuation is generally associated with a higher risk of relapse in anxiety disorders in the general population [58]. Although studies specifically examining the relapse of ARD in pregnant women discontinuing antidepressants are lacking, a meta-analysis in pregnant patients suffering from depression showed a trend toward relapse following discontinuation [59].

#### Postpartum effects of SSRIs on the infant

Neonatal adaptation syndrome may occur in about 30% of the infants after birth subjected to third trimester in utero exposure to SSRI [60]. It is generally a mild, self-limiting, behavioral syndrome emerging from the first days after birth and disappearing within 2 weeks after birth [60, 61]. Symptoms vary from the occurrence of gastrointestinal disturbances to respiratory distress, motor tone abnormalities, or body temperature instability. Susceptibility to neonatal adaptation syndrome appears to be dose-dependent, with higher maternal SSRI doses associated with poorer neonatal adaptation [61].

Because of the potential instability of the infant, mothers are generally advised to give birth in a hospital, which is standard in most countries, with pediatric assessment and extra monitoring available [62]. Generally, with supportive care and extra observation, neonatal adaptation syndrome can be managed; internationally, the length of observation ranges from 12 h to 3 days [60, 62]. On rare occasions, a neonatal intensive care unit (NICU) admission is necessary to support the infants during the neonatal adaptation syndrome as was described using data from a Swedish national registry (*n* = 262 329) [52].

SSRIs pass into breast milk in very small amounts. Paroxetine, fluvoxamine, and sertraline produce undetectable plasma levels. The highest infant plasma levels have been found for fluoxetine, citalopram, and venlafaxine (in some infants up to more than 80% for fluoxetine and 30% venlafaxine of the assumed adult dosage) [63]. While this may lead to non-specific symptoms in the baby (such as poor sleep, crying, or poor growth), most babies are not affected [63, 64].

To conclude, SSRI use can lead to side-effects in mother and fetus, but these are generally mild and transient. Most commonly used SSRIs are well studied and are considered safe [44, 45]. Sertraline is preferred as SSRI in treatment-naive women when starting an antidepressant during pregnancy, considering the smallest risk for PPH in infants and the relatively small amounts passing into breast milk [37, 44, 45, 63, 65]. Switching from an effective SSRI is discouraged, even when the currently used agent is a non-preferred SSRI [62].

### Preventive strategies to mitigate the vulnerability for anxiety in children of anxious parents

In clinical practice, we notice that, when patients with ARD become parents, they often seek advice on how to prevent their children from developing ARD. Although research on these strategies is limited, some preventive parenting strategies and interventions have been identified and described below.

#### Promote trust and autonomy

Based on a meta-analysis of 17 experimental studies including children aged 30 months to 18 years, which showed that parental verbal threat information can increase children’s immediate fear responses to novel stimuli, parents are advised to be mindful of frequent threat-based warnings (e.g., “watch out,” “don’t touch this,” “be careful”) [66]. The effect of the parental verbal threat information was large and independent of the child’s age. These findings come from controlled settings in which children were exposed to verbal information about unfamiliar stimuli, and therefore do not imply a universal recommendation for all parenting contexts. Reducing unnecessary threat-focused messages may help support children’s autonomy and confidence [66].

#### Preventive interventions aimed at families, children, or parents

A few researchers have examined preventive interventions for children with anxious parents. However, promising results have been found with two family-based, cognitive–behavioral and psychoeducational interventions, reducing the likelihood of developing ARD in children with 26% (31% in control group versus 5% in intervention group) and 8.5% (60% in control group versus 51.5% in intervention group), respectively [67, 68]. These interventions consist, among other components, of psychoeducation on identifying and reducing anxiety, cognitive restructuring, in vivo desensitization, problem-solving techniques, and parenting strategies, such as contingency management and the promotion of child independence and autonomy. Implementation of these short-term interventions, involving the participation of both parents, is needed [69]. Although standardized anxiety treatments, solely addressing parental symptoms, may also indirectly benefit children, a recent systematic review identified no studies on this topic [70]. An effect can be expected, since a meta-analysis on depression indicates that psychotherapy for parents with depression has a positive effect on their children’s mental health [71]. Further research is needed due to the heightened risk of ARD among children [72].

**Table:** 

Clinical recommendations	
Psychotherapy during pregnancy	
1	Pregnant women often prefer psychotherapy over medication
2	Psychotherapy (including exposure therapy and EMDR) seems effective in treating pregnant women with ADR
3	Non-pharmacological interventions in pregnant women with ARD appear to be safe for the fetus, although adverse effects with a very low incidence cannot be ruled out
4	EMDR for mothers experiencing fear of childbirth seems to have no adverse effects on pregnancy or fetus outcomes
5	Therapists should be aware of their own reservations about using exposure therapy, the first choice treatment in ARD
Pharmacotherapy during pregnancy	
1	For treatment-naïve pregnant patients, sertraline is the preferred SSRI of choice. Switching of an effective SSRI during pregnancy is discouraged, even when using a non-preferred SSRI
2	Side-effects of maternal SSRI use are generally mild and transient for the newborn
3	Serious adverse effects include persistent pulmonary hypertension for the fetus and postpartum hemorrhage and pre-eclampsia for the mother. All have a slightly increased relative risk; the absolute risk remains small
Parenthood	
1	Guide parents in minimizing verbally expressing (presumed) threat information
2	If available, recommend interventions to prevent the onset of an anxiety-related disorder in children of anxious parents, such as family-based interventions

## Data Availability

No datasets were generated or analyzed during the current study.
